# In-Hospital Clinical Outcomes in Patients with Fragility Fractures of the Lumbar Spine, Thoracic Spine, and Pelvic Ring: A Comparison of Data before and after Certification as a DGU^®^ Geriatric Trauma Centre

**DOI:** 10.3390/medicina57111197

**Published:** 2021-11-03

**Authors:** Markus Laubach, Laura Christine Gruchow, Tobias Hafner, Filippo Migliorini, Matthias Knobe, Frank Hildebrand, Miguel Pishnamaz

**Affiliations:** 1 Department of Orthopaedics, Trauma and Reconstructive Surgery, RWTH Aachen University Hospital, 52074 Aachen, Germany; laura.christine.gruchow@rwth-aachen.de (L.C.G.); thafner@ukaachen.de (T.H.); fmigliorini@ukaachen.de (F.M.); fhildebrand@ukaachen.de (F.H.); mpishnamaz@ukaachen.de (M.P.); 2 Department of Orthopaedic and Trauma Surgery, Lucerne Cantonal Hospital, 6004 Lucerne, Switzerland; matthias.knobe@luks.ch

**Keywords:** fragility fracture, elderly, geriatric trauma centre, orthogeriatric co-management

## Abstract

*Background and Objectives*: The implementation of orthogeriatric co-management (OGCM) reflects the demand for interdisciplinary collaborations due to the increasing comorbidities of geriatric trauma patients. This study aimed to assess clinical in-hospital outcomes in lumbar spine, thoracic spine, and pelvic ring fragility fracture patients before and after the implementation of a Geriatric Trauma Centre (GTC) certified by the German Trauma Society (DGU^®^). *Materials and Methods*: In this observational, retrospective cohort study, geriatric trauma patients (>70 years of age) were stratified into either a pre-GTC group (hospital admission between 1 January 2012 and 31 December 2013) or a post-GTC group (hospital admission between 1 January 2017 and 31 December 2018). Patients’ pre-injury medical complexity was measured by ASA class (American Society of Anaesthesiologists classification), the use of anticoagulant medication, and the ACCI (Age-adjusted Charlson Comorbidity Index). Outcome parameters were patients’ in-hospital length of stay (LOS) and mortality rates, as well as new in-hospital findings and diagnoses. Further, the necessity of deviation from initial management plans due to complications was assessed using the Adapted Clavien–Dindo Scoring System in Trauma (ACDiT score of ≥1). *Results*: Patients in the post-GTC group (*n* = 111) were older (median age 82.0 years) compared to the pre-GTC group (*n* = 108, median age 80.0 years, *p* = 0.016). No differences were found in sex, body mass index, ASA class, or ACCI (all *p* > 0.05). Patients in the post-GTC group used vitamin K antagonists or direct oral anticoagulants more frequently (21.3% versus 10.8%). The incidence of non-surgical treatment and mortality was comparable between groups, while LOS tended to be shorter in the post-GTC group (7.0 days versus 9.0 days, *p* = 0.076). In the post-GTC group, the detection of urinary tract infections (UTI) increased (35.2% versus 16.2%, *p* = 0.001), and the delirium diagnoses tended to increase (13.0% versus 6.3%, *p* = 0.094), while an ACDiT score of ≥1 was comparable between groups (*p* = 0.169). *Conclusions*: In this study including lumbar spine, thoracic spine, and pelvic ring geriatric fragility fractures, patients in the post-GTC group were more medically complex. More UTIs and the tendency for increased delirium detection was observed in the post-GTC group, likely due to improved diagnostic testing. Nonetheless, the necessity of deviation from initial management plans (ACDiT score of ≥1) was comparable between groups, potentially a positive result of OGCM.

## 1. Introduction

The incidence of fragility fracture patients is rising, especially in more economically developed countries. Fragility fractures occur mainly in the aging population, causing a high disease burden [1,2,3]. It is estimated that 30–50% of all people over 50 years old will suffer at least one osteoporosis-associated vertebral body fracture [4]. Further, in industrialised countries, geriatric pelvic ring fractures, which are associated with long-term reduced mobility and quality of life [5], have significantly increased over the past 50 years and are predicted to increase by 2.4-fold by the year 2030 [6]. Notably, medical complexity of geriatric trauma patients has significantly increased during the last decade [7]. In particular, the elderly have an overall greater risk of in-hospital complications [8] and a threefold higher mortality rate as compared to younger patients [9,10].

Geriatric patients are particularly vulnerable, and due to their specific characteristics and demands, they require multidisciplinary perioperative care to prevent in-hospital complications [11]. Further, the use of vitamin K antagonists (VKAs) or direct oral anticoagulants (DOACs) is common in the elderly and has been associated with significantly increased posttraumatic morbidity and mortality [12,13]. Therefore, in 1978, the Queens Medical Centre in Nottingham was the first to establish an “Orthogeriatric Unit” in order to promote shared responsibility between orthopaedic surgeons and doctors from additional disciplines, such as geriatricians, to achieve optimal acute care for older patients with proximal femur fractures. Thereby, the short- and long-term sequalae associated with in-hospital complications may be reduced, aiming to avoid morbidity, loss of autonomy, need for long-term institutional care, and mortality [14]. In fact, a systematic review showed that when compared to standard care, geriatric hip fracture patients treated with orthogeriatric co-management (OGCM) such as that provided within a Geriatric Trauma Centre (GTC) had reduced mortality, fewer hospital-acquired pressure ulcers, and superior functional outcomes [15,16]. Overall, interdisciplinary collaborations with improved teamwork and team effectiveness [17] better met elderly patients’ special needs during the entire perioperative phase [18].

A recently published bibliometric analysis of fragility fractures showed that the majority of research groups investigating clinical management have focused on hip fracture patients [19]. In contrast to hip fractures, for which prompt surgery is associated with improved outcomes, non-surgical management is indicated in a relevant proportion of spine and pelvic ring fragility fracture patients. As longer non-surgical therapies can lead to increased mortality, reduced mobility, and a loss of social independence [20], these patient groups may particularly benefit from OGCM.

The aim of this study was to compare the clinical outcomes of lumbar spine, thoracic spine, and pelvic ring geriatric fracture patients before and after the structural implementation of OGCM after certification as a GTC by the German Trauma Society (DGU^®^). Due to demographic changes, we hypothesised that the medical complexity of geriatric trauma patients has increased, and that these patients may benefit from a GTC DGU^®^.

## 2. Materials and Methods

The study design is an observational retrospective cohort study in accordance with the STROBE criteria [21]. The clinical setting was a level 1 trauma centre. Data acquisition was based on an analysis of patients’ electronic medical records (EMRs). Extraction of data was done automatically when possible, and missing or incomplete data sets were assessed manually by reviewing patient charts. The study was approved by the local Ethics Committee (EK 284/16) and is registered with the Institutional Centre for Translational and Clinical Research (no. 16-666).

### 2.1. Certification as a DGU^®^ Geriatric Trauma Centre (GTC)

The clinic was certified as a DGU GTC in January 2015 following an independent audit process. Certification as a GTC includes embedding OGCM into structural and processual requirements in accordance with the guidelines of the DGU [22,23], as previously described in detail by our group [24]. Briefly, geriatric trauma patients receive routine consultation with a geriatrician in an interdisciplinary ward round twice per week. Further, once per week, during a team conference, all cases are discussed in an interdisciplinary manner, and patient-specific treatment plans are defined. In addition to participation in team conferences, representatives from nursing, occupational therapy, physiotherapy, and case management are actively involved in the clinical treatment process throughout the entire hospital stay. To establish an individual risk profile, each trauma patient aged > 70 years undergoes an Identification of Seniors at Risk Screening (ISAR screening) upon hospital admission. In accordance with the quality criteria of a GTC DGU^®^, an ISAR score ≥2 indicates a recommendation for OGCM. The implementation phase for the GTC took place from 2015 to 2016 as the structural and processual requirements of the DGU criteria catalogue for the certification process were successfully realised, including all interdisciplinary collaborations [25].

### 2.2. Study Population

Lumbar spine, thoracic spine, and pelvic ring fracture patients aged > 70 years were included in this study. An age of >70 years for eligibility for treatment with OGCM follows the current DGU recommendations for GTC certification [22] and is in line with the German Society for Geriatrics’ definition of geriatric patients [26]. Patients who were readmitted were not included in order to prevent statistical fallacies due to repeated measurements. In addition, patients with polytrauma (Injury Severity Score ≥ 16), those discharged from the intensive care unit, those with an elective admission for implant removal, and those with malignancy-associated fractures were not considered. As the implementation phase was likely associated with a “transition effect” involving minor structural and processual adjustments, we included geriatric trauma patients who were admitted between 1 January 2012 and 31 December 2013 (stratified to the pre-GTC group), or between 1 January 2017 and 31 December 2018 (stratified to the post-GTC group).

### 2.3. Demographic Characteristics and Medical Complexity of the Study Population

All patient data used in this study were retrieved anonymously from EMRs, partially automatically and partially manually. Basic demographic data including sex and body mass index were retrieved. The definition of complex elderly in this study was based on biological determinants of health [27], for which the term medical complexity is used hereafter. The medical complexity of patients was assessed using the ISAR screening, ASA class (American Society of Anaesthesiologists classification), use of anticoagulant medication, prefracture dementia, and the Age-adjusted Charlson Comorbidity Index (ACCI). The ISAR screening consists of six items formulated as closed questions [28] concerning a patient’s need for assistance, acute changes in their need for assistance, previous hospitalisations in the last six months, their visual ability and cognitive impairment, and the daily use of six or more different medications. The score can range from 0 to 6 points. A score of ≥2 points is considered positive, indicating an increased likelihood of need for geriatric intervention [29]. In addition, the ASA class of prefracture physical status was quantified to assess overall preoperative health, ranging from 1 (healthy person) to 5 (moribund) [30]. This classification has prognostic value for perioperative morbidity and mortality [31]. In this study, ASA class was obtained from a pre-anaesthesia evaluation in the case of surgical procedures; in the case of non-surgical procedures, ASA score was mainly determined retrospectively based on recorded comorbidities [32]. Individual anticoagulant medications including vitamin K antagonists (VKAs), direct oral anticoagulants (DOACs), antiplatelet drugs (APDs), and heparin and heparinoid anticoagulants were reported. Further, prefracture diagnosis of dementia was recorded. Information on the presence and severity of prefracture comorbidities was further quantified using the ACCI. The ACCI is a predictive index for mortality based on 19 items including cardiopulmonary, neurological, and tumour diseases. To adjust for patient age, comorbidities and age are combined into one index score [33]. The assessment was carried out retrospectively using recorded prefracture morbidities.

### 2.4. In-Hospital Courses before and after GTC Certification

The in-hospital courses of the present study population included details regarding individual fracture sites, the number of patients non-surgically treated, the in-hospital lengths of stay in days (LOS), and mortality. Fracture sites were, as per the inclusion criteria, for the lumbar and thoracic spine as well as the pelvic ring. For multiple injuries, the main diagnosis reported in the EMR was the decisive factor in assigning the fracture site. Individual patient treatment courses, including estimated time until discharge, were planned in an interdisciplinary manner to achieve early geriatric rehabilitation after acute trauma treatment.

### 2.5. New in-Hospital Findings and Diagnoses

New findings and diagnoses occurring during hospitalisation were retrieved from recordings of patients’ diagnosis-related groups (DRGs) [34] and from discharge letters. A comparison of admission and discharge reports also revealed new findings and diagnoses that were determined during hospital stays. The new reported in-hospital findings and diagnoses included delirium; diagnoses grouped into cardiological, pulmonary, and gastrointestinal categories; urinary tract infections (UTIs); anaemia; and electrolyte disorders.

### 2.6. Adapted Clavien–Dindo Scoring System in Trauma (ACDiT)

The Adapted Clavien–Dindo Scoring System in Trauma (ACDiT) was used to grade the severity of new in-hospital findings and diagnoses. This scoring system allows for the grading of new, post-traumatic in-hospital findings and the diagnosis of both surgically and conservatively treated patients. New in-hospital findings and diagnoses were classified from grades ranging from 0 to Vb based on their consequences, ranging from no deviation from the initial management plan to pharmacological intervention, transfer to intensive care units, and death [35]. This score allowed for pre- and post-GTC comparison of the severity of new in-hospital findings and diagnoses. The results of the ACDiT scoring system were dichotomised into groups of ACDiT 0 (no required deviation from the initial management plan) and ACDiT ≥ 1 (deviation from initial management plan required) in accordance with Naumann et al. [35].

### 2.7. Statistical Analysis

The demographic characteristics and medical complexity of the study population, their in-hospital courses before and after GTC certification, and new in-hospital findings and diagnoses, as well as the ACDiT scores of the study population, are provided using descriptive statistics. Data on categorical variables are presented as percentages (%). A Pearson chi-squared test was used to compare categorical variables with more than five expected observations, and Fisher’s exact test was applied for categorical variables with less than five. The Shapiro–Wilk test for the normal distribution of data was also performed. The data are presented as medians with interquartile ranges (IQRs) for continuous, non-normally distributed variables compared between groups using the Mann–Whitney U test. Normally distributed variables were summarised as means and standard deviations (SD), and an independent Student’s *t*-test was performed for group comparison. Two-tailed *p*-values of <0.05 were considered significant. IBM SPSS Statistics (version 26; Armonk, New York, NY, USA) was used for statistical analyses.

## 3. Results

### 3.1. Demographic Characteristics and Medical Complexity of the Study Population

Descriptive data on the demographic characteristics and medical complexity of the study population are summarised in Table 1. In total, 219 geriatric patients with lumbar spine, thoracic spine, and pelvic ring fractures were included and stratified to either the pre-GTC group (*n* = 111) or the post-GTC group (*n* = 108). Patients in the post-GTC group were older compared to the pre-GTC group (median age of 82.0 versus 80.0 years, *p* = 0.016). No differences were observed between groups regarding sex (*p* = 0.135) or body mass index (*p* = 0.078). In this study, ISAR screening was performed in 90.74% of patients (post-GTC group), and 69.4% reached the cut-off score (≥2), proving the need for OGCM in the majority of our patient population. No significant difference was observed between groups for ASA class (*p* = 0.475), with more patients classified as ASA 3 or higher (pre-GTC group 71.2%, post-GTC group 75.9%) than ASA 1 or 2 (pre-GTC group 28.8%, post-GTC group 24.1%). Significant differences in anticoagulant medication (*p* = 0.007) were observed, with the post-GTC group accounting for more VKAs and DOACs (21.3% versus pre-GTC 10.8%) and less APDs (16.7% versus pre-GTC 32.4%). Diagnosis of dementia did not differ between groups (*p* = 0.279). A median ACCI in the pre-GTC group of 5 points (IQR 5–6) and a median ACCI of 6 points (IQR 5–7) in the post-GTC group were observed (*p* = 0.261).

### 3.2. In-Hospital Course before and after GTC Certification

The fracture sites differed between the pre-GTC and post-GTC groups (*p* = 0.026). The fracture sites in the pre-GTC group were more often the thoracic spine (37.8% versus post-GTC 23.1%) versus the pelvic ring (22.5% versus 36.1%). LOS tended (*p* = 0.076) to be shorter post-GTC (7.0 days, IQR 5.0–11.8) when compared to pre-GTC (9.0 days, IQR 6.0–13.0), and in-hospital mortality rate had a persistent low level in both groups (Table 2).

### 3.3. New in-Hospital Findings and Diagnoses

Delirium tended to be diagnosed more frequently in the post-GTC group (13.0% versus 6.3%, *p* = 0.094). Further, patients in the post-GTC group were diagnosed with UTIs more often (35.2% versus 16.2%, *p* = 0.001). No differences were observed for new cardiological, pulmonary, or gastrointestinal diagnoses; anaemia; or electrolyte disorders (Table 3).

### 3.4. Adapted Clavien–Dindo Scoring System in Trauma (ACDiT)

**Figure 1:**
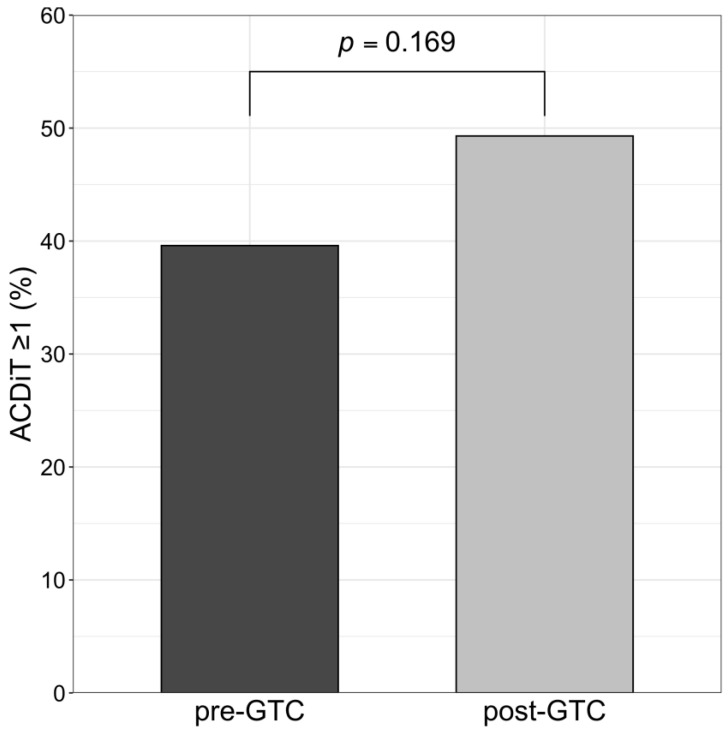
Necessity of deviation from initial management plans due to in-hospital course complications as indicated by the Adapted Clavien–Dindo Scoring System in Trauma (ACDiT). The percentage of patients with an ACDiT ≥ 1 was not significantly different between groups. GTC, Geriatric Trauma Centre.

We observed that new in-hospital findings and diagnoses necessitated deviation from patients’ initial management plans (grade ≥ 1 ACDiT) in 39.6% of patients in the pre-GTC group and in 49.3% of patients in the post-GTC group (*p* = 0.169, Figure 1).

## 4. Discussion

The World Health Organisation (WHO) has declared the period from 2020 to 2030 to be the “Decade of Healthy Aging”. One of the four key aims (“calls to action”) is to “deliver integrated care and primary health services that are responsive to the needs of older people” [36]. The care of patients with fragility fractures particularly challenges healthcare professionals because the fracture patients’ physical conditions differ from those of younger patients [6,25]. In particular, premorbid frailty and multiple co-existing conditions in the older trauma population may impact patient health during in-hospital treatment [37]. To meet the specific requirements associated with geriatric trauma patients, interdisciplinary collaborations consisting of orthopaedic surgeons and geriatricians can be certified by the DGU as GTCs after demonstrating the required processual and structural characteristics. This study sought to investigate the relationship of the implementation of a GTC with the outcomes of geriatric lumbar spine, thoracic spine, and pelvic ring fracture trauma patients. Our main findings are as follows:

1. LOS tended to decrease, and persistent low level of in-hospital mortality was observed in both groups, despite increased medical complexity in the post-GTC group (characterised by significantly increased age and higher use of VKAs and DOACs).
2. The detection of UTIs increased, and a tendency toward more delirium diagnoses in the post-GTC group was observed.
3. Despite the increased medical complexity of geriatric patients, we observed no difference in the necessity for deviations from initial management plans (grade ≥ 1 ACDiT).

We observed a tendency toward shorter LOS in the post-GTC group. This is in contrast with a recent observational study based on health insurance claim data from 58,001 patients treated in German hospitals [16]. Rapp et al. [16] observed a longer LOS in patients who were treated with OGCM and argued that this observation was due to the fact that these patients had already received rehabilitative treatment during their index hospital stays [16]. However, after acute care, trauma patients in the present cohorts were typically transferred to affiliated hospitals specialising in early geriatric rehabilitative treatment. Therefore, the shorter LOS in the post-GTC group may, rather, be interpreted as being due to earlier mobilisation and transferability to institutes with highly specialised geriatric rehabilitative treatment options. Nonetheless, according to previous studies, the interdisciplinary collaboration in GTCs itself seems also to be associated with reduced LOS, preventing complications, and enabling more patients to return home safely [7,38,39].

Further, the mortality rate in both of our study groups was low (pre-GTC 0.9%, post-GTC 3.7%). In contrast with previous studies reporting on mortality in patients treated in a GTC [40,41], we did not include polytrauma patients or those discharged from the intensive care unit. These strict inclusion criteria may be the reason for the lower in-hospital mortality compared with previous studies, which report mortality before implementation of OGCM between 5.1% and 9.5% and after implementation of OGCM between 3.4% and 6.5% [40,41]. The present study is more comparable to a study with a similar design that assessed the effect of comprehensive orthogeriatric care compared to standard orthopaedic care, also excluding patients who had suffered a pathological fracture or had multiple traumas, and similar mortality rates were herein observed (in-hospital mortality both groups 2.2%) [42]. Overall, however, implementation of a GTC has generally shown not to result in a significant reduction in mortality [24,40,43], which is in line with our study results.

We interpret the higher rates of UTIs and delirium in the post-GTC group as being due to improved detection rather than actual increased incidence. Particularly, improvements in diagnostic tools such as laboratory tests, screening tools, and clinical scoring systems [44], as well as a higher awareness associated with the interdisciplinary management, might have led to more new findings in the post-GTC group. UTIs are the second-most common infection in the geriatric population, with associated morbidity and mortality, but in older patients, typical findings for infectious diseases are not always observed [45,46]. Therefore, as required deviations from initial management plans were comparable between groups, it is conceivable that OGCM not only facilitates the early detection of UTIs, but also provides support for the optimal treatment of such complications. Further, in line with a previous study including a triggered geriatric consult model, we achieved improved diagnosis and documentation of delirium [47]. While often underdiagnosed in the absence of proactive monitoring [48], delirium is associated with long-term cognitive decline and increased mortality in both surgical and nonsurgical patients [49,50]. Our higher detection rate of delirium after the implementation of OGCM is in line with previous studies [39,51,52]. Therefore, as early detection of delirium is crucial [53], we interpret these findings as being favourable to GTC because the improved detection of UTIs and delirium in turn facilitates improved treatment.

The ACDiT includes a useful trauma endpoint that is non-binary, clinically meaningful, and patient-centred [35]. To the best of our knowledge, with the application of the ACDiT, we are the first to utilise a validated score incorporating newly emerged findings and diagnoses to assess the need for deviation from initial management plans. Pre-existing comorbidities and/or frailty in geriatric trauma patients have been associated with higher in-hospital costs [54]. On the other hand, OGCM of geriatric patients with hip fractures was associated with significant decreases in hospital charges [43]. Despite increased medical complexity in patients, we observed no increase in necessity of deviation from initial management plans (grade ≥ 1 ACDiT) between both groups. Therefore, it is conceivable that further increases in healthcare costs may be avoided with interdisciplinary OGCM in a GTC. However, prospective studies that quantify direct and indirect health care costs are required to confirm this assumption by observing cohorts of patients treated both surgically and non-surgically.

We note strengths and limitations. A strength of the present study is that increased comparability of routine clinical treatment was achieved by avoiding potential “transition effects” associated with the implementation phase of a GTC. Further, information bias was minimised by manually retrieving missing or incomplete patient data from EMRs when automatic retrieval was incomplete. While a consistent definition of a medically complex older person is lacking, we have focused on biological determinants of health. Inclusion of multiple determinants of health and their interrelationships might better address the concept of medical complexity [55]. However, due to the lack of information in hospital administrative data, the opportunity to explore the interrelationships between social support, multimorbidity, and clinical outcomes was not in the scope of this study. Another limitation of this study is its retrospective data collection. As opposed to prospective studies, this reduces the reliability of the data collected and limits the observation period, which was restricted here to the in-hospital stay. Further, this study was conducted in a level 1 trauma centre with potential admission of more vulnerable geriatric patients. Therefore, it was possible that selection bias of patients was induced. An ISAR score of ≥2 is the cut-off value for treatment in a GTC DGU. The majority of the patients in the post-GTC group reached this cut-off score. Nonetheless, patient selection in the post-GTC group was independent of ISAR score. We anticipated that patients with an ISAR score of <2 would also be affected by the structural and processual improvements associated with GTC DGU certification and, therefore, included these patients to ensure comparability between groups.

## 5. Conclusions

In geriatric cases of lumbar, thoracic, and pelvic ring fractures, LOS tended to decrease after GTC certification, and a persistent low level of in-hospital mortality was observed post-GTC compared to pre-GTC. Despite increased medical complexity and detection of UTIs as well as a trend towards more delirium diagnoses in the post-GTC group, no difference was observed in the number of patients who required deviation from initial management plans. Future prospective studies may be helpful to evaluate causality and confirm our findings.

## Figures and Tables

**Table 1:** Demographic characteristics and medical complexity of study population ^a^.

	Pre-GTC (*n* = 111)	Post-GTC (*n* = 108)	*p* Value
Age (years), median (IQR)	80.0 (76.0–84.0)	82.0 (77.3–86.8)	0.016 ^b^
Sex (female), *n* (%)	86 (77.5)	74 (68.5)	0.135
BMI (kg/m^2^), mean (SD) ^†^	25.8 (4.5)	24.6 (4.2)	0.078 ^b^
ISAR score (IQR)		3.0 (1.0–4.0) ^‡‡^	
ASA class, *n* (%)			
ASA 1 and ASA 2	32 (28.8)	26 (24.1)	0.475
ASA 3 and higher	79 (71.2)	82 (75.9)	
Anticoagulant medication, *n* (%) ^††^			
None	44 (39.6)	46 (42.6)	0.007
VKAs or DOACs	12 (10.8)	23 (21.3)	
APDs	36 (32.4)	18 (16.7)	
Heparin and heparinoids	16 (14.4)	7 (6.5)	
Dementia, *n* (%) ^‡^	24 (21.6)	17 (15.7)	0.279
Age-adjusted Charlson Comorbidity Index, median (IQR) ^‡^	5.0 (5.0–6.0)	6.0 (5.0–7.0)	0.261 ^c^

APD, Antiplatelet drugs; ASA class, American Society of Anesthesiologists classification; BMI, body mass index; DOAC, direct oral anticoagulants; IQR, interquartile range; ISAR, Identification of Seniors at Risk; Geriatric Trauma Centre, GTC; SD, standard deviation; VKA, Vitamin K antagonists. ^†^ Data missing for 42 patients in the pre-GTC group and for 31 patients in the post-GTC group. ^‡^ Data missing for one patient in the post-GTC group. ^††^ Data missing for 3 patients in the pre-GTC group and for 14 patients in the post-GTC group. ^‡‡^ No ISAR screening conducted in 10 of the 108 patients (9.26%). ^a^ Pearson’s chi-squared test unless otherwise specified. ^b^ Independent Student’s *t*-test. ^c^ Mann–Whitney U test.

**Table 2:** In-hospital courses before and after certification as Geriatric Trauma Centre (GTC) ^a^.

	Pre-GTC (*n* = 111)	Post-GTC (*n* = 108)	*p* Value
Fracture site			
Thoracic spine, *n* (%)	42 (37.8)	25 (23.1)	0.026
Lumbar spine, *n* (%)	44 (39.6)	44 (40.7)	
Pelvic ring, *n* (%)	25 (22.5)	39 (36.1)	
Non-surgical treatment, *n* (%)	47 (42.3)	56 (51.9)	0.159
LOS (days), median (IQR)	9.0 (6.0–13.0)	7.0 (5.0–11.8)	0.076 ^b^
Mortality, *n* (%)	1 (0.9)	4 (3.7)	0.208 ^c^

IQR, interquartile range; LOS, length of in-hospital stay. ^a^ Pearson’s chi-squared test unless otherwise specified. ^b^ Mann–Whitney U test. ^c^ Fisher’s exact test.

**Table 3:** New in-hospital findings and diagnoses upon patient admission ^a^.

	Pre-GTC (*n* = 111)	Post-GTC (*n* = 108)	*p* Value
Delirium, *n* (%)	7 (6.3)	14 (13.0)	0.094
Cardiological, *n* (%)	13 (11.7)	9 (8.3)	0.406
Pulmonary, *n* (%)	17 (15.3)	19 (17.6)	0.649
Gastrointestinal, *n* (%)	9 (8.1)	4 (3.7)	0.168
Urinary tract infection, *n* (%)	18 (16.2)	38 (35.2)	0.001
Anaemia, *n* (%)	20 (18.0)	14 (13.0)	0.302
Electrolyte disorder, *n* (%)	37 (33.3)	27 (25.0)	0.175

Geriatric Trauma Centre, GTC. ^a^ Pearson’s chi-squared test unless otherwise specified.

## Data Availability

The data that support the findings of this study are available from the corresponding author (M.L.) upon reasonable request.
